# The ambulatory care of patients with post-acute sequelae of COVID-19

**DOI:** 10.1007/s43999-023-00020-y

**Published:** 2023-02-22

**Authors:** Christoph Strumann, Wolfgang C. G. von Meißner, Paul-Georg Blickle, Jost Steinhäuser

**Affiliations:** 1 Institute of Family Medicine, University Hospital Schleswig-Holstein, Campus Lübeck, Ratzeburger Allee 160, Lübeck, 23538 Germany; 2 Hausärzte am Spritzenhaus, Family Practice, Baiersbronn, Germany

**Keywords:** SARS-CoV-2, Covid-19, Post-Covid, Long-covid, Post-acute sequelae of COVID-19 (PASC), Primary care, Germany

## Abstract

**Background:**

There is an increasing number of patients that do not make a rapid or full recovery from a SARS-CoV-2 infection, the Coronavirus Disease 2019 (COVID-19) and suffer from the so-called “long-COVID” or post-acute sequelae of COVID-19 (PASC). The long-term implications for health services are expected to be substantial. The objective of this analysis was to estimate the utilization of outpatient services from primary and secondary care. Further, we evaluated the multidisciplinary ambulatory care management of PASC patients in Germany.

**Methods:**

All members of the Physician network “MEDI Baden-Württemberg e.V.”, i.e., 1,263 primary care physicians (PCPs) and 1,772 specialists working in secondary care were invited to participate in a questionnaire surveying routine data regarding the general care situation at the physician practice level of patients suffering from PASC. Bivariate analyses were applied to consider potential differences between primary and secondary care.

**Results:**

In total, 194 physicians participated in this survey (response rates of 9.6% (primary care) and 4.1% (secondary care). On average, each physician treated 31.9 PASC patients. Most PASC patients (61.2%) had three or more long-COVID symptoms. On average, 10.6 PASC patients visited a physicians’ practice per quarter. The additional consulting effort for treating PASC patients was 34.1 min (median: 20 min) and higher in primary care. Most PCPs (71.1%) integrated secondary care in the treatment of their PASC patients. Less than half of the PASC patients (42.0%) sought secondary care with a referral from primary care. 5.7 patients visited the physicians’ practices per week, who were concerned about suffering from PASC without any following medical confirmation. This caused an average additional effort for the physicians of 17.5 min per visit. There were no differences between rural and urban areas.

**Conclusion:**

Our results reveal that there is a substantial additional consulting effort for treating PASC patients that is especially high in primary care. The additional consulting effort results from the consultation of a particular high number of patients that are concerned about suffering from PACS without a following medical confirmation. To guarantee a high quality and adequate provision of care for a potentially further increasing number of concerned patients, the ambulatory health services should be strengthened and adequately compensated.

**Supplementary Information:**

The online version contains supplementary material available at 10.1007/s43999-023-00020-y.

## Introduction

With the ongoing SARS-CoV-2 pandemic, an increasing number of patients is observed that do not make a rapid or full recovery from a Coronavirus Disease 2019 (COVID-19). Patients report persistent symptoms or new symptoms arise after recovery for weeks or month encompassing several organs and systems as well as nonspecific symptoms [1]. The range and the number of reported symptoms present a broad spectrum of manifestations including pulmonary sequelae, neurologic disorders, mental health disorders, functional mobility impairments, and general and constitutional symptoms [2, 3].

Estimated prevalence statistics of the so-called long-COVID or post-acute sequelae of COVID-19 (PASC) vary between 10 and 20% among patients with mild courses of the disease [4] and are larger among patients with a higher severity [5–7]. In the UK, around 1.8 million people (2.8% of the total population) had recently reported to suffer from symptoms persisting for more than 4 weeks after their (confirmed or suspected) infection with SARS-CoV-2 as of 3 April 2022; 44% or 13% of these individuals had been infected at least one year or two years ago, respectively [8]. Since PASC has been also observed among patients with mild and even asymptotic infections, it cannot be ruled out that breakthrough or re-infections could also cause long-term effects as observed for example for the Gamma variant of SARS-CoV-2 [9]. Data from UK suggest that the risk of developing PASC was reduced by around 50% in those who were double-vaccinated [10]. Based on data of US veterans, a previous vaccination reduced the risk of developing PASC after infection by 15% [11]. Given the emergence of the Omicron variant in November 2021 with an increased global spread, a rise of patients suffering from PACS can be expected [12]. First results suggest that 4.5% of vaccinated people experience long COVID after an Omicron infection, in comparison with 10.8% for patients infected with the Delta variant [13].

Although approaches to define PASC have not been completed yet [3], the long-term implications for health services are expected to be substantial [14]. Given the lack of evidence how to care the involved patients and the broad spectrum of reported health problems highlight the need for holistic integrated services for patients with persistent symptoms [15]. Primary care is usually the first port of call for people with a broad variety of health problems in many countries. Therefore, primary care providers are in a unique position to provide and coordinate care for their patients suffering from PASC [16].

In Germany, patients have free provider choice and direct access to medical specialists of any sector [17]. However, about 85% of the patients infected with SARS-CoV-2 were cared for mostly by primary care physicians (PCPs) in community practices [18]. Regardless of the recent trend that an increasing number of post-COVID outpatient clinics are opening and offering PASC patients a first point of contact, the vast majority of patients with post-COVID syndrome were cared by ambulatory private practices [19]. However, the implied burden for the physicians caused by an increased utilization of outpatient services is not evaluated yet.

In this analysis, we estimated the utilization of outpatient services from primary and secondary care and evaluated the multidisciplinary ambulatory care management of PASC patients in Germany.

## Methods

### Questionnaire and data collection

This cross-sectional analysis was performed online between 3rd and 31st December 2021 using Surveymonkey©. The questionnaire was sent to all members of the physician network “MEDI Baden-Württemberg e.V.”, i.e., 1,263 PCPs and 1,772 secondary care physicians (SCPs). The rationale for the questionnaire was to gather data describing the routine ambulatory care of PASC patients in Germany until December 2021. The questionnaire was not formally validated but piloted for clearness and understandability. It included 18 items about the care situation at the physician practice level (see Supplement 1 in the Appendix for more details). The physicians should estimate the number of treated patients that have had a SARS-CoV-2 infection and the number of PASC patients from the beginning of the pandemic to the date of the survey. Further, they should provide information about the number of PASC patients that experienced three or more symptoms. In the questionnaire, a list of symptoms to be specific for PACS according to related literature [1, 20] have been provided^1^. The physicians were also asked whether they are working in rural or an urban area depending on their subjective categorization.

### Statistical analysis

To characterize the care situation in Germany while also considering potential differences between primary and secondary care as well as differences between rural and urban areas, we tabulated the specific variables for the respective subgroups. Differences between subgroups were tested for statistical significance by employing the Mann-Whitney U-test due to high skewness of the data. Statistical analyses were performed with STATA 15 (StataCorp LLC, College Station, TX, USA).

## Results

### Sample characteristics

In total, 194 physicians participated in this survey (overall response rate of 6.4%, PCP: 9.6% and SCP: 4.1%). The mean age of the participants was 58 years, 30.2% were female and 51.1% worked in an urban area (see Table 1). Most participants were providing primary care (62.4%). This share was higher in rural areas. On average, the surveyed PCPs treated 271.9 patients in 53.7 h per week. Regarding secondary care, the number of treated patients was 136.8 with 49.3 h per week.

**Table 1:** Sample Characteristics, n (%)

Variable		working in a	
	total	rural area	urban area
	*n* = 194 (100%)	*n* = 92 (48.9%)	*n* = 96 (51.1%)
*Socio-demographics*			
Age (mean)	58.0	58.5	57.5
Female	57 (29.4)	28 (30.4)	29 (30.2)
Male	132 (68.0)	64 (69.6)	67 (69.8)
missing gender information	5 (2.6)	0 (0.0)	0 (0.0)
*Specialty*			
Primary Care	121 (62.4)	67 (72.8)	52 (54.2)
Trainee^a^	3 (1.5)	1 (1.1)	2 (2.1)
Family medicine^a^	67 (34.0)	42 (45.7)	23 (24.0)
General internal medicine^a^	28 (14.2)	11 (12.0)	17 (17.7)
General practitioner^a^	2 (1.0)	1 (1.1)	1 (1.0)
Pediatricians^a^	10 (5.1)	3 (3.3)	7 (7.3)
missing primary care specialty information^a^	13 (6.7)	9 (9.8)	2 (2.1)
Secondary Care	73 (37.6)	25 (27.2)	44 (45.8)
Pulmonology^a^	5 (2.5)	1 (1.1)	4 (4.2)
Cardiology^a^	6 (3.1)	3 (3.3)	2 (2.1)
Neurology^a^	14 (7.1)	5 (5.4)	9 (9.4)
Psychiatry^a^	13 (6.6)	6 (6.5)	7 (7.3)
other^a^	42 (21.3)	14 (15.2)	28 (29.2)
missing secondary care specialty information^a^	0 (0.0)	0 (0.0)	0 (0.0)
*Average Working characteristics*			
Primary Care			
working hours per week	53.7	53.3	54.2
number of patients per week	271.9	284.1	256.0
patient contact time in minutes	12.2	10.6	14.3
Secondary Care			
working hours per week	49.3	52.2	48.0
number of patients per week	136.8	155.9	126.4
patient contact time in minutes	21.6	19.4	23.0

^a^ The numbers do not to sum up to the total number of primary or secondary care, since multiple choices were allowed within primary and secondary care, respectively

### Subgroup analysis

In Table 2, the mean, the median and the standard deviation are shown for all physicians and for the subgroups of primary and secondary care as well as for the subgroups of rural and urban areas. For most variables, there were substantial differences between the mean and the median. The average total number of treated patients with a SARS-CoV-2 infection per physician to date was 165.9, while the median was 80. Both statistics (mean and median) were higher for PCPs in comparison with SCPs (216.1 (PCP) vs. 82.4 (SCP), $p<$0.1%). On average, each physician treated 31.9 PASC patients with a median of 6. While the average number was significantly higher for secondary care (22.9 vs. 47.4), the median was lower (10 vs. 4). Regarding the median number of PASC patients per specialist group obtains a heterogeneous distribution: the median pulmonologist treated 300, cardiology: 25, neurology: 10, psychiatry: 10 and others: 1 (these numbers are not shown in Table 2).

**Table 2:** Subgroup analysis (mean [median] (standard deviation))

Variable			Primary vs. Secondary care			rural vs. urban area		
		all	Primary	Secondary		rural	urban	
	missing obs.	*n* = 194 (100%)	*n* = 121 (62.4%)	*n* = 73 (37.6%)	*p*-value	*n* = 92 (48.9%)	*n* = 96 (51.1%)	*p*-value
patients with a SARS-CoV-2 infection (in total)	7	166.7 [80] (231.8)	216.1 [150] (227.0)	82.4 [10] (216.6)	< 0.001	179.9 [50] (269.2)	153.7 [100] (186.9)	n.s.
PASC patients (in total)	10	31.9 [6] (92.9)	22.9 [10] (37.7)	47.4 [4] (145.1)	0.020	42.6 [5] (125.9)	20.3 [10] (30.7)	n.s.
PASC patients with 3 + symptoms	16	14.7 [4] (30.3)	12.3 [4] (20.7)	19.3 [4] (42.6)	n.s.	16.0 [3] (36.0)	13.3 [4] (23.0)	n.s.
percentage of PASC patients with 3 + symptoms (in %)	33	61.2 [66.7]	55.3 [55.0]	73.9 [88.0]	< 0.001	61.5 [65.9]	61.4 [66.7]	n.s.
PASC patients visiting the practice (per quarter)	12	10.6 [4] (23.6)	8.2 [4] (17.5)	14.9 [3] (31.7)	n.s.	14.0 [3] (31.8)	7.1 [4] (9.4)	n.s.
additional consulting effort per patient treating PASC patients (min)	16	34.1 [20] (49.6)	43.0 [20] (57.8)	17.1 [15] (18.6)	< 0.001	36.8 [15] (58.7)	31.5 [20] (39.1)	n.s.
with 1–2 symptoms (min)	51	26.6 [20] (35.4)	32.5 [20] (41.7)	15.7 [10] (13.3)	< 0.001	27.9 [15] (41.8)	25.1 [20] (27.1)	n.s.
with 3 + symptoms (min)	47	55.3 [30] (118.4)	62.0 [30] (83.6)	43.1 [20] (164.4)	< 0.001	58.9 [30] (145.8)	51.6 [30] (80.7)	n.s.
PASC patients admitted to hospital	11	1.3 [0] (3.2)	1.5 [0] (3.5)	0.9 [0] (2.3)	n.s.	1.5 [0] (3.9)	1.0 [0] (2.2)	n.s.
percentage of PASC patients admitted to hospital (in %)	34	8.5 [0]	8.3 [0]	9.0 [0]	n.s.	10.1 [0]	7.0 [0]	n.s.
PASC patients referred to ambulatory care centers	15	2.7 [0] (11.0)	3.3 [1] (13.1)	1.4 [0] (4.5)	0.001	2.0 [0] (4.6)	3.4 [0] (15.1)	n.s.
percentage of PASC patients referred to ambulatory care centers (in %)	38	15.4 [0.0]	16.3 [5.6]	13.4 [0.0]	0.034	15.6 [0.0]	15.5 [5.0]	n.s.
Patients who visit the practice with the concern about suffering from PASC without medical confirmation (per week)	12	5.7 [2] (9.7)	5.8 [2] (9.2)	5.4 [1] (10.6)	0.012	4.9 [2] (9.1)	6.4 [2] (10.3)	n.s.
additional consulting effort of these patients (min)	24	17.5 [15] (21.0)	20.3 [15] (23.5)	12.4 [10] (14.2)	< 0.001	15.7 [12.5] (21.8)	19.6 [15] (20.1)	0.012
Ratio between patients without and with medical confirmation of PASC^a^	34	18.8 [6.5] (35.1)	20.0 [6.5] (32.6)	16.2 [3.8] (40)	0.004	16.1 [6] (33.4)	21.4 [6.5] (36.8)	n.s.

n.s. not significant (at the 5% level)

^a^ To compute the ratio, first, the number of PASC patients visiting the practice (per quarter) is divided by the average number of weeks per quarter (i.e., 13). Second, the number of patients who visit the practice with the concern about suffering from PASC without medical confirmation (per week) is divided by the previous computed number

Most PASC patients (61.2%) had three or more post-COVID symptoms. This proportion was higher for patients treated in secondary care. On average, 10.6 PASC patients visited the physicians’ practices per quarter of the year. The additional consulting effort for treating PASC patients was reported, on average, with 34.1 min (median: 20 min). The duration was significantly lower for secondary care. The mean number of PASC patients that were hospitalized due to their suffering of persistent symptoms was 1.3 (8.5%). On average, each physician referred 2.7 PASC patients (15.4%) to ambulatory care centers (median: 2%). A closer look on the distribution of this variable revealed that 52% of the physicians had not referred any PASC patient to these centers (45% (primary care) vs. 70% (secondary care)), while 12% referred more than half of their PASC patients (11% (primary care) vs. 13% (secondary care)) (these numbers are also not shown in Table 2). In urban areas, the number of referred patients was higher (3.4 (urban) vs. 2.0 (rural)). However, the difference in the absolute as well as in the relative number were not significant.

5.7 patients who were concerned about suffering from PASC without following medical confirmation visited the physicians’ practices per week, causing an average additional effort of 17.5 min. In relation to the patients with a medical confirmation about their suffering of PASC, the number of patients without a medical confirmation was, on average, about 18.8 times higher (median: 6.5). This ratio was larger in primary care.

There are no significant differences in the utilization of outpatient services between rural and urban areas, except for the additional consulting effort of patients who were concerned about suffering from PASC without following medical confirmation. The average additional effort is significantly higher in urban areas (19.6 min (urban) vs. 15.7 min (rural), $p=$1.2%).

### Referral pattern

Most PCPs (71.1%) integrated secondary care in the treatment of their PASC patients (Table 3). These were mostly referred to pulmonologist (39.2%) and cardiologists (30.8%). Less than half of the PASC patients (42.0%) sought secondary care with a referral from primary care. These percentages were higher in pulmonology (62.0%) and cardiology (65.0%) and were the lowest in other specialist groups (28.0%) including orthopedics and trauma surgery, otorhinolaryngology, urology, and gynecology and obstetrics.

**Table 3:** Referrals

	Primary Care		Special Care
	Primary care physicians with co-treatment by secondary care	PASC patients referred to secondary care	PASC patients with referrals from primary care
	n (%)	mean (%)	mean (%)
total	86 (71.1)	NA	31.7 (42.0)
Pulmonology	75 (62.0)	4.6 (39.2)	235.0 (62.0)
Cardiology	70 (57.9)	3.7 (30.8)	14.5 (65.0)
Neurology	48 (39.7)	2.8 (22.3)	16.8 (45.3)
Psychiatry	29 (24.0)	1.8 (13.9)	18.1 (45.6)
other	14 (11.6)	NA	1.5 (28.0)

*NA* Not applicable

Differences in the referral patterns between rural and urban areas had not been detected (see Table S3 in the Appendix).

## Discussion

In this analysis, we estimated the utilization of outpatient services of patients that report persistent symptoms longer than 8 weeks after their SARS-CoV-2 infection (PASC). Our data reveal that there are substantial differences between primary and secondary care. Instead, differences between rural and urban areas seems to be negligible.

In our survey, the PCPs reported that 10% of all their patients with a known SARS-CoV-2 infection suffered from PASC. This number is similar to international statistics estimating the prevalence of PASC to vary between 10 and 20% among patients with mild courses of the disease [4]. While primary care has higher numbers of patients with a SARS-CoV-2 infection, the number of PASC patients is higher in secondary care as well as the percentage of PASC patients suffering from three or more symptoms. In contrast, the additional consulting effort per patient is higher in primary care. Since in Germany, about 85% of the patients infected with SARS-CoV-2 were cared for mostly by PCPs [18], PCPs might also be the first contact for PASC patients. An analysis of German routine data found that 76% of the PASC patients were treated in primary care only [21]. Further, most of the PASC patients have been before their SARS-CoV-2 infection undergoing medical treatment for various (mostly chronic) illnesses. The most common pre-existing conditions in 2020 relate to major common diseases such as back pain (41%) and hypertension (37%), but also include mental health problems, e.g., depression (18%), somatoform disorders (19%), and stress and adjustment disorders (13%) [19]. Due to the broad spectrum of reported health problems and the emotional consequences experienced by people with PASC [22, 23], the recognition of the condition and an empathic validation of the patient’s experience is crucial to effectively manage PASC [14]. The PCP typically know their patients and is aware about their life circumstances enabling to coordinate and personalize the recovery plan [14]. The preselected patients are referred from primary care to further special treatment, most of them to pulmonology [2]. However, only 42% of the PASC patients received a referral from their PCP. In comparison with common referral rates for patients in Germany, this number is rather high. In 2016, only 15.5% of the patients visiting a specialist have had a referral from a PCP due to the design of the health care system [24].

There is a high variation between physicians referring their PASC patients to ambulatory care centers that are specialized on post-COVID treatment. While some physicians referred more than half of their patients, more than half of the physicians have not referred any patient to these centers. This might be explained by the lacking availability of such centers that are mostly located at university hospitals. Further, critics on these clinics include long waiting-lists, difficulties to train clinicians, barriers to access for patients living in rural areas, absence of integrated multidisciplinary rehabilitation and long-term funding sustainability. Instead, a complete care model should integrate primary care, rehabilitation services and specialized clinics for medical assessment [25]. Primary care should be strengthened to address the additional efforts that are needed to manage the care of this rising group of patients [16, 26].

As our data suggest, not only the treatment of patients that suffer from PACS has resulted in additional effort. Instead, much more patients without a PACS confirmation are concerned about suffering of PACS and visit the physicians’ practices. This can be observed especially in primary care and in urban areas. Based on an internet survey on the health status of the French population, only half of the participants stating that they had already contracted COVID-19, had have a confirmation by a blood test for antibodies [27]. Further, a significant association with subjective belief of having previously had COVID-19 was found in 15 of 18 possible long-COVID symptoms. Due to a dominance in attention of the pandemic, many people seem to be inclined to attribute current health complaints to a previous infection with SARS-CoV-2. The fact that fatigue, shortness of breath, concentration problems, sleep disturbances, gastrointestinal complaints, anxiety and depression are generally of high prevalence and can also have other causes seems to be not taken into consideration enough. To what degree this is due to stigmatization of some diseases compared with PACS should be the matter of future studies.

As shown in our results, the additional consulting effort of the ambulatory health care provider for treating patients that are concerned about suffering from PACS leads to an increase in consultations, especially in primary care. As these reasons for encounters are not specific reimbursed, primary care face a double burden: high patient volume and time-consuming consultations. Even though there is a definitive diagnosis for PASC (i.e., U09.9!, Post-COVID-19 condition), the treatment of some of the concerned patients will not result in a diagnosis relevant to billing, but will still require time for consultation.

### Strengths and limitations

A strength of this analysis is that it relies on the reporting of primary and secondary care physicians located in rural and urban areas from the federal state of Baden-Württemberg. This allows a rather broad assessment of the experiences of physicians managing SARS-CoV-2 infected patients with persistent symptoms in Germany. However, the representativeness of the findings could be impaired by a selection bias or response bias [28], due to the low response rate of 6.4% that resulted in a rather small sample size of surveyed physicians (*n* = 194). The age of the sampled physicians (58 years) is, however, similar to the average age of private practice physicians in Baden-Württemberg (55 years). The percentage of female physicians is smaller than the respective average percentage of women in private practice (30% vs. 46%) [29]. Due to the possibility of de-identification of the surveyed physicians, we have not collected geographical data to determine the urbanity of the physicians’ location. Instead, we based the definition of rural and urban areas solely on the physicians’ subjective categorization.

To analyze the referral behavior of the PCPs, it might be important to elaborate on the degree of PACS, since referrals are not necessary if the PCP can already treat the symptoms. Additionally to the question of how many PACS patients the PCP generally have referred to other health care providers, they were also asked for the number of referred patients separated for PACS patients with only one symptom and PACS patients with two or more symptoms allowing to discriminate between both groups. However, the number of missing values was too high for this analysis.

The data collection is based on the physicians’ experiences that is sensitive to noise. For the individual physician, the memory might highly depend on other sources we cannot control for [30]. However, since the physicians answered independently and we are only interested in average statistics like the mean or median number of treated PASC patients, the noise problem might not play an important role in our analysis. Further, in this study, only the physician perspective was considered to evaluate the implied burden for the physicians caused by an increased utilization of outpatient services. Future research should also take into account data extracted from the patient perspective to confirm the results of this study.

## Conclusion

In this analysis, we estimated the utilization of outpatient services from private practices and evaluated the multidisciplinary ambulatory care management of SARS-CoV-2 patients in Germany that suffer from persistent symptoms. Our results reveal that there is a substantial additional consulting effort for treating PASC patients. This additional consulting effort is especially high in primary care and results also from the consultation of a particular high number of patients that are concerned about suffering from PACS without a medical confirmation. To guarantee a high quality and adequate provision of care for a potentially further increasing number of concerned patients, the ambulatory health services should be strengthened and adequately compensated. Especially, a strong primary care that is the first contact of care provides holistic, person-focused and comprehensive care over time, and coordinates specialized care is needed to manage the care of this rising group of patients.

### Supplementary Information

**Additional file 1.** Post-COVID syndrome survey.

**Additional file 2: Table S3.** Referrals.

## Data Availability

Making the data available to third parties is not covered by the ethics vote.

## Abbreviations

COVID-19: Coronavirus Disease 2019
PASC: post-acute sequelae of COVID-19
PCP: primary care physician
SCP: secondary care physician

## Footnotes

1 This list includes: fatigue, impaired taste and smell, impaired performance, shortness of breath at rest, shortness of breath on exertion, headache, impaired activity, cough, sleep disorders, depressed mood, anxiety symptoms, post-traumatic stress disorder, general pain, altered breathing pattern, impaired concentration and memory, obsessive-compulsive behavior, hair loss, skin rash, stress, paralysis and sensory disturbances, dizziness, nausea, diarrhea, loss of appetite, tinnitus, earache, loss of voice, limb pain, chest pain, cardiac arrhythmias (palpitations), palpitations (tachycardia).
