# Supplementary material for: The ambulatory care of patients with post-acute sequelae of COVID-19
Source: Res Health Serv Reg. 2023 Feb 22;2:4. doi: 10.1007/s43999-023-00020-y (PMC9943722; doi:10.1007/s43999-023-00020-y)
Supplement: Supplementary file 1 — Additional file 1. Post-COVID syndrome survey. [file 43999_2023_20_MOESM1_ESM.pdf]

Post-COVID syndrome survey

Dear colleague,

The majority of patients in Germany who are infected with the coronavirus are not treated in hospital, but in the ambulatory sector. This is also true for the treatment of patients who report persistent complaints after their infection (such as fatigue, taste and odor disturbances, impaired performance, etc. [1]), the so-called post-COVID syndrome (PCS). However, data of this routine care are hardly visible so far.

Therefore, we would like to ask you to answer the following questions about the treatment effort for PCS patients. It will take a maximum of five minutes to complete the questionnaire.

The survey will be anonymous, and a positive ethics vote has been obtained.

Best regards

[1] Other symptoms include: Shortness of breath at rest, Shortness of breath under stress, Headache, Activity limitation, Cough, Sleep disturbances, Depressive mood, Anxiety symptoms, Post-traumatic stress disorder, General pain, Altered breathing pattern, Impaired concentration and memory, Obsessive-compulsive behavior, Hair loss, Skin rash, Stress, Paralysis and sensory disturbances, Dizziness, Nausea, Diarrhea, Loss of appetite, Tinnitus, Earache, Loss of voice, Limb pain, Chest pain, Cardiac arrhythmias (palpitations), Palpitations (tachycardia)

Sources:
- Nalbandian et al. (2021). Post-acute COVID-19 syndrome. Nat Med 27, 601–615. https://doi.org/10.1038/s41591-021-01283-z
- Koczulla et al. (2021). S1-Leitlinie Post-COVID/Long-COVID. AWMF-Register Nr. 020/027. https://www.awmf.org/uploads/tx_szleitlinien/020-027l_S1_Post_COVID_Long_COVID_2021-07.pdf

For the following questions, please provide an estimate.

1. How many patients with corona infection have you treated so far?

2. What is the total number of patients who report persistent symptoms longer than 8 weeks after their corona infection (post-COVID syndrome) that you have cared for?

3. In your experience, how many of these patients have three or more post-COVID symptoms (see above)?

4. How much additional care do you estimate is required for patients suffering from post-COVID syndrome? (in minutes per patient)
a) In general
b) If applicable, for one or two post-COVID symptoms
c) If applicable, for three or more post-COVID symptoms

5. In relation to patients who are concerned about suffering from post-COVID syndrome without medical confirmation:
a) How many of this patient group do you treat per week?
b) What do you estimate the (additional) consulting effort to be? (in minutes per patient)

6. On average, how many times per quarter does a patient with post-COVID syndrome visit your practice?
a) General
b) If applicable, for one to two post-COVID symptoms
c) If applicable, for three or more post-COVID symptoms

7. How many of your patients in total have had to be hospitalized for post-COVID syndrome?
a) General
b) If applicable, for one to two post-COVID symptoms
c) If applicable, for three or more post-COVID symptoms

8. How many of your patients had to be referred to a specialized outpatient clinic because of post-COVID syndrome?
a) General
b) If applicable, for one to two post-COVID symptoms
c) If applicable, for three or more post-COVID symptoms

Please answer the following questions about your medical practice.

9. What is your average weekly working time? (in hours per week)

10. What is the average number of patients you treat per week?

11. What do you estimate your average patient contact time to be? (in minutes)

12. Your gender
male
female
diverse

13. Your age in years

14. Where do you operate?
in the city
in rural area

15. Do you provide primary care?
Yes
No

Primary care physicians

16. You are working as
Physician in postgraduate training
Specialist in family medicine/ General Practitioner
Specialist in internal medicine
Practical physician
Specialist in pediatrics and adolescent medicine

17. How many of your patients with post-COVID syndrome have been treated or are being treated by physicians from other specialties?
a) General
b) If applicable, for one to two post-COVID symptoms
c) If applicable, for three or more post-COVID symptoms

18. In total, how often have you involved physicians from the following specialties in the treatment of patients with post-COVID syndrome?

	General	If applicable, for one to two post-COVID symptoms	If applicable, for three or more post-COVID symptoms
Pulmonology			
Cardiology			
Neurology			
Psychiatry			

Further, namely:

Secondary care physicians

16. In which specialty do you work? As a physician in
Postgraduate training
Anesthesiology
Anatomy
Occupational medicine
Ophthalmology
Biochemistry
General surgery
Vascular surgery
Cardiac surgery
Pediatric and Adolescent Surgery
Orthopedics and Trauma Surgery
Plastic, Reconstructive and Aesthetic Surgery
Thoracic surgery
Visceral surgery
Gynecology and obstetrics
Otorhinolaryngology
Skin and venereal diseases
Human genetics
Hygiene and environmental medicine
Internal Medicine
Internal Medicine and Angiology
Internal Medicine and Endocrinology and Diabetology
Internal Medicine and Gastroenterology
Internal Medicine and Hematology and Oncology
Internal Medicine and Cardiology
Internal Medicine and Nephrology
Internal Medicine and Pneumology
Internal Medicine and Rheumatology
Pediatrics and Adolescent Medicine
Child and Adolescent Psychiatry and Psychotherapy
Laboratory Medicine
Microbiology, Virology and Infectious Disease Epidemiology
Oral and maxillofacial surgery
Neurosurgery
Neurology
Nuclear Medicine
Public Health
Neuropathology
Pathology
Clinical Pharmacology
Pharmacology and Toxicology
Phoniatrics and Pediatric Audiology
Physical and rehabilitative medicine
Physiology
Psychiatry and Psychotherapy
Psychosomatic medicine and psychotherapy
Radiology
Forensic Medicine

Radiotherapy
Transfusion medicine
Urology
Psychological psychotherapy (incl. child and adolescent psychotherapy)
Other, namely:

17. How many patients with post-COVID syndrome (in %) have been referred to your practice by a primary care physician?

Both groups of physicians

18. Do we have forgotten an important aspect? We look forward to your comments.

Thank you very much for your support!
