# Supplementary material for: The ambulatory care of patients with post-acute sequelae of COVID-19
Source: Res Health Serv Reg. 2023 Feb 22;2:4. doi: 10.1007/s43999-023-00020-y (PMC9943722; doi:10.1007/s43999-023-00020-y)
Supplement: Supplementary file 2 — Additional file 2: Table S3. Referrals. [file 43999_2023_20_MOESM2_ESM.docx]

Table S3: Referrals

|  | Primary care physicians with co-treatment by secondary care, n (%) | | | | PASC patients referred to secondary care, mean (%) | | | | PASC patients with referrals from primary care, mean (%) |
| --- | --- | --- | --- | --- | --- | --- | --- | --- | --- |
|  | total | urban | rural | p-value | total | urban | rural | p-value |  |
| total | 86 (71.1) | 40 (76.9) | 46 (68.7) | 0.318 | NA | NA | NA | NA | 31.7 (42.0) |
| Pulmonology | 75 (62.0) | 34 (65.4) | 41 (61.2) | 0.639 | 4.6 (39.2) | 5 (37.6) | 4 (41.2) | 0.194 | 235.0 (62.0) |
| Cardiology | 70 (57.9) | 32 (61.5) | 38 (56.7) | 0.596 | 3.7 (30.8) | 4.3 (32.2) | 3 (29.3) | 0.223 | 14.5 (65.0) |
| Neurology | 48 (39.7) | 22 (42.3) | 26 (38.8) | 0.699 | 2.8 (22.3) | 3.3 (25.1) | 2.4 (19.5) | 0.465 | 16.8 (45.3) |
| Psychiatry | 29 (24.0) | 15 (28.8) | 14 (20.9) | 0.316 | 1.8 (13.9) | 2.3 (15.8) | 1.3 (12.1) | 0.700 | 18.1 (45.6) |
| other | 14 (11.6) | 8 (15.4) | 6 (9.0) | 0.280 | NA | NA | NA | NA | 1.5 (28.0) |

NA: not applicable
